# Scientific Discovery Framework Accelerating Advanced Polymeric Materials Design

**DOI:** 10.34133/research.0406

**Published:** 2024-07-08

**Authors:** Ran Wang, Teng Fu, Ya-Jie Yang, Xuan Song, Xiu-Li Wang, Yu-Zhong Wang

**Affiliations:** The Collaborative Innovation Center for Eco-Friendly and Fire-Safety Polymeric Materials (MoE), National Engineering Laboratory of Eco-Friendly Polymeric Materials (Sichuan), State Key Laboratory of Polymer Materials Engineering, College of Chemistry, Sichuan University, Chengdu 610064, China.

## Abstract

Organic polymer materials, as the most abundantly produced materials, possess a flammable nature, making them potential hazards to human casualties and property losses. Target polymer design is still hindered due to the lack of a scientific foundation. Herein, we present a robust, generalizable, yet intelligent polymer discovery framework, which synergizes diverse capabilities, including the in situ burning analyzer, virtual reaction generator, and material genomic model, to achieve results that surpass the sum of individual parts. Notably, the high-throughput analyzer created for the first time, grounded in multiple spectroscopic principles, enables in situ capturing of massive combustion intermediates; then, the created realistic apparatus transforming to the virtual reaction generator acquires exponentially more intermediate information; further, the proposed feature engineering tool, which embedded both polymer hierarchical structures and massive intermediate data, develops the generalizable genomic model with excellent universality (adapting over 20 kinds of polymers) and high accuracy (88.8%), succeeding discovering series of novel polymers. This emerging approach addresses the target polymer design for flame-retardant application and underscores a pivotal role in accelerating polymeric materials discovery.

## Introduction

Organic polymer materials, widely integrated into various sectors of the national economy and daily life, stand out as the most abundantly produced materials [1–3]. However, unlike metallic and inorganic non-metallic materials, organic polymers possess a flammable nature [4–6], making them prone to ignition and contributing to fire incidents that lead to both human casualties and property losses. Bestowing flame retardancy upon organic polymer materials emerges as a paramount solution [7–9], acting at the root to mitigate the risk of fires and playing an indispensable role. Despite this, there is still a grand challenge [10,11] for target polymer design because existing research on flame-retardant mechanisms has largely been conducted in simulated environments [12,13], not in real fire environments. Current polymer design still relies on empirical design [14,15] rather than scientific understanding. Consequently, the insights gained from such studies fall short of guiding effective flame-retardant design, rendering the conclusions ineffective for target design. Addressing this dilemma is crucial for elevating the scientific foundation of flame retardancy in organic polymer materials.

Recently, high-throughput experiments [16–18], combining the latest technological innovations in automation and computer science [19–21] into materials synthesis [22] and characterization [23,24], have become a revolutionary accelerator to research novel materials in industry and academia. Among them, hardware robots [25] or analyzers for high-throughput characterization can complete multiple batches of realistic experiments in a short time sequence to markedly reduce the material discovery period. Recently, the material genome method [26–28] is derived from artificial intelligence technology based on big data [29,30], predictive models [31,32], machine learning algorithms, and chemical space [33,34]; it systematically filters a large number of possible structures and discovers a range of high-performance materials [35–37] with target properties by the artificial intelligence program in computer platforms. The new paradigm from heterogeneous toolkits (high-throughput experiments with machine-learning-centered science) working together has become an encouraging potential for material design.

In particular, polymeric materials withstanding fire environments compel a selective thermal breakdown of chemical bonds within polymer chains, giving rise to chaotic combustion reactions with intricate physicochemical phenomena. State-of-the-art fire-safety materials often rely on their predominant intermediates [38–40]—containing aromatic, halogen, phosphorus, and nitrogen motifs—to terminate the combustion chain reactions by the flame-retardant roles in the gas phase or condensed phase manner [41,42]. Yet, obtaining these large amounts of transient intermediates by current experimental methods proves impractical due to harsh acquisition environments. Furthermore, unlike inherent properties (thermal properties, dielectric properties, thermal stability, mechanical behavior, etc.) that are primarily determined by the structure sequence itself, the flame-retardant performance is not solely related to the structure sequence, and more strongly related to fire conditions. Adding to the complexity, the synthesis and subsequent testing for new polymeric materials are time-consuming and expensive processes. These dilemmas always lead to failure design of high-performance materials.

**Fig. 1.**
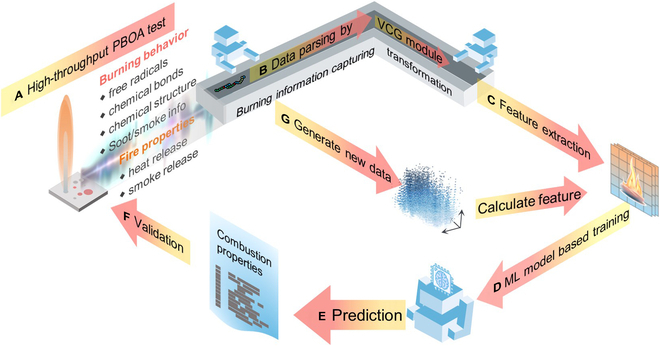
The scientific discovery framework integrated heterogeneous tools. In a single discovery workflow, (A) high-throughput instrument PBOA tests both the flame retardancy and the real burning behavior; (B) accumulative data updating the VCG module to resolve more combustion information; (C) combustion information being embedded as the feature; (D) training a machine learning model; (E) predicting flame retardancy using the trained ML model; (F) validating the predicted results; (G) generating new structures using data parsed by the VCG module. The closed-loop process can iterate and gradually improve the efficiency and accuracy of the scientific discovery framework.

In response, we propose a scientific framework beyond the empirical paradigm of polymer design for target polymer design, integrating the in situ burning analyzer, virtual reaction generator, and material genomic model. In Fig. 1, the ideal framework includes a novel polymer burning operando analyzer (PBOA) that can real-time, in situ, and high-throughput capture intermediate information, enabling a deeper understanding of the fundamentals of polymer burning; a virtual combustion generator (VCG) obtains the combustion reaction rules of polymers and their flame-retardant systems from massive combustion data, bringing a faster-run and lower-cost virtual analysis while extracting more intermediate information for modeling; an efficient feature engineering strategy, embedding combustion intermediates information into molecular representations to more accurately predict flame-retardant properties. Polymer discovery and synthesis tasks successfully validate the reliability of the framework, illustrating its potential to facilitate the scientific discovery of the applied polymers with high flame retardancy.

## Results

### Polymer burning operando analyzer

**Fig. 2.**
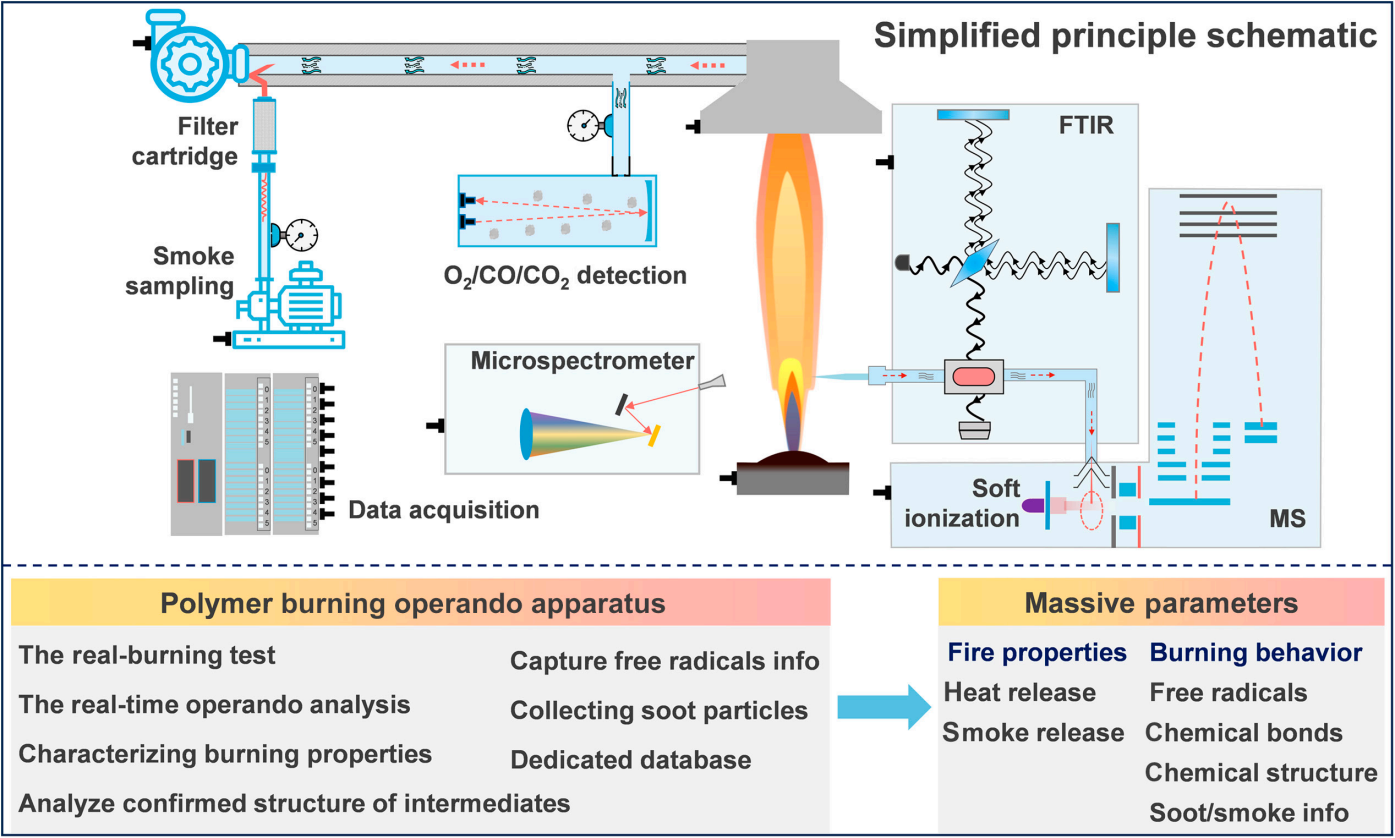
Polymer burning operando analyzer.

As a first step in proving the discovery framework, the PBOA (Fig. S1) in situ tests the burning processes based on spectroscopy principles (Fig. 2; emission spectrum in the ultraviolet–visible region, Fourier transform infrared spectrum, soft ionization mass spectrometry, laser emission spectrum, etc.) after systematically resolving the multi-point sampling challenge in burning conditions (Table S1) and further acquires massive parameters (chemical bonds, chemical structure, diatomic free radicals, soot particles, heat release, smoke release, mass loss). Meanwhile, an online database for applied polymers (*PolymData*, https://polymdata.scu.edu.cn) for high-throughput retrieving spectroscopy data and confirming intermediate parameters is also released online in a structured data format (Fig. S2).

The analysis method for in situ testing of the burning processes is as follows: (a) igniting and continuously burning materials to support the real-burning reaction conditions; (b) collecting combustion parameters as completely as possible by steel sampling rings, quartz needles, and nonintrusive optical sampling; (c) characterizing the burning properties by producing heat and smoke release data; (d) measuring the signals including wavenumbers, mass-to-charge ratio, wavelength of emission spectra, intensities; (e) rapidly indexing out the chemical bonds, exact structure, and diatomic free radicals information from *PolymData*. Visualized testing data are exhibited in Fig. S3. Unlike the conventional methods that are simply incompatible with researching the real burning processes, PBOA, as a novel analyzer to synchronously obtain the burning property and behavior, provides an important characterized method; it also brings the basis for a big-data-driven polymer discovery.

### Virtual combustion generator

**Fig. 3.**
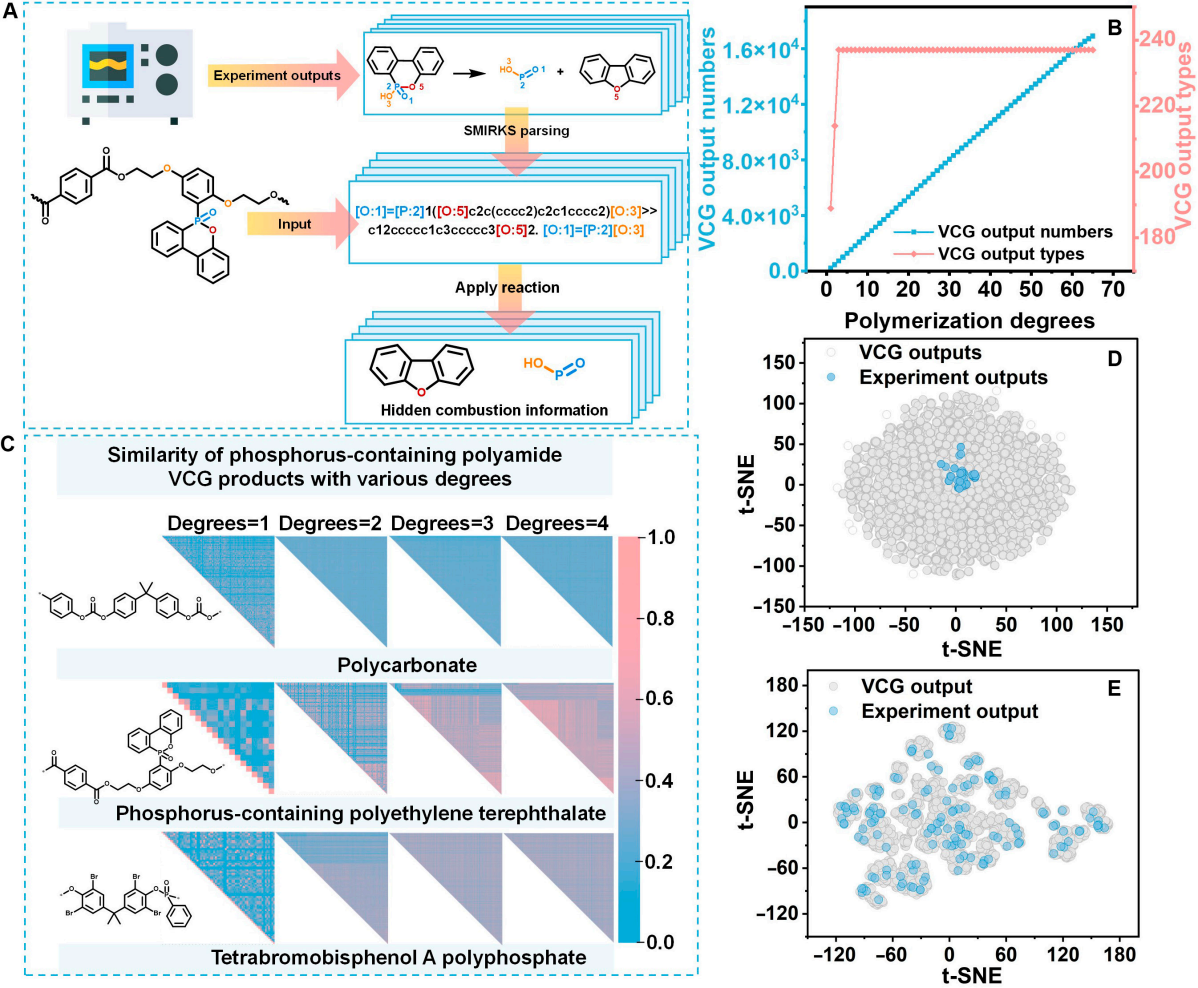
The construction process and data distribution comparison of the VCG. (A) The construction processes. (B) Quantities and types change with degrees of polymers as input. (C) Chemical diversity analysis of the representing example at different polymerization degrees. (D) Experimental and VCG output (polymer chains with the polymerization degree of 3) comparison intermediates. (E) Comparison between experimental and VCG output data of polycarbonate.

Generally, although the created PBOA instrument can obtain as much information on combustion intermediates as possible, some hidden intermediate information is still missing due to the complex combustion. Therefore, we create a VCG module and give its constructed processes as shown in Fig. 3A, including collecting the reaction rules from real combustion data, parsing to reaction templates based on these rules, and inputting polymer sequences to resolve more hidden combustion information. Figure S4 exhibits collected reaction rules for semi-polyesters, just as an example and merges the same types of reactions. In Fig. S5, summarized combustion templates (44 sets) are given using SMIRKS reaction transform language into chemical reaction templates. As a result, the molecule matrix is obtained, which includes all products for each site where the reaction takes place (each row corresponds to the particular reaction site). This process generates product atoms mapped by the reactive core, and unmapped atoms on SMIRKS products are created and added to the resulting products, and the corresponding new bonds (from the unmapped atom to other atoms) are created as well. Some products formed by unmapped atoms are intermediates that are hardly captured in realistic experiments.

Before inputting the polymer sequence into the reaction templates, we explore the effects of chain molecular weight. The quantities and types of intermediates generated by polymer sequences with varying degrees of polymerization are compared. As shown in Fig. 3B, quantities linearly increase with increasing polymerization degree, but the types of intermediates tend to stabilize after the polymerization degree of 3, illustrating that polymers with a higher molecular weight produce more intermediates but the types tend to converge. Additionally, chemical diversity among these intermediates at different polymerization degrees is assessed by the molecular similarity analysis using Morgan4 molecular fingerprints. Here, we choose 3 kinds of polymers as displayed examples. In Fig. 3C, although the number of intermediates increases linearly with increasing polymerization degree, the Tanimoto distance between new VCG intermediates in high polymerization degree is always in the range of 0.2 and 0.8. In particular, the trends of similarity distributions basically remain consistent after a polymerization degree of 3, indicating that the chemical diversity converges with increasing polymerization degree. That is, the inputting polymer sequence with the polymerization degree of 3 is the optimal selection.

Notably, VCG can calculate more intermediate information on burning processes than the experimental results as shown in Fig. 3D (the blue and gray dots represent the experiment and VCG results, respectively), in which the chemical space of intermediates obtained by executing VCG into the polymer dataset has covered that from burning experiments. Taking polycarbonate as an example in Fig. 3E, 629 intermediates (listed in Table S2) from VCG not only include experiment results (142 kinds of compounds, blue dots) but also reveal more hidden information (such as O=C=O, CCC, O=CO, and other active small-molecule structures) hardly captured by experimental characterization. Thereby, VCG can capture massive hidden intermediates in the combustion process beyond the experiments, thus providing more features for establishing the following genomic model.

### Machine learning genomic models

**Fig. 4.**
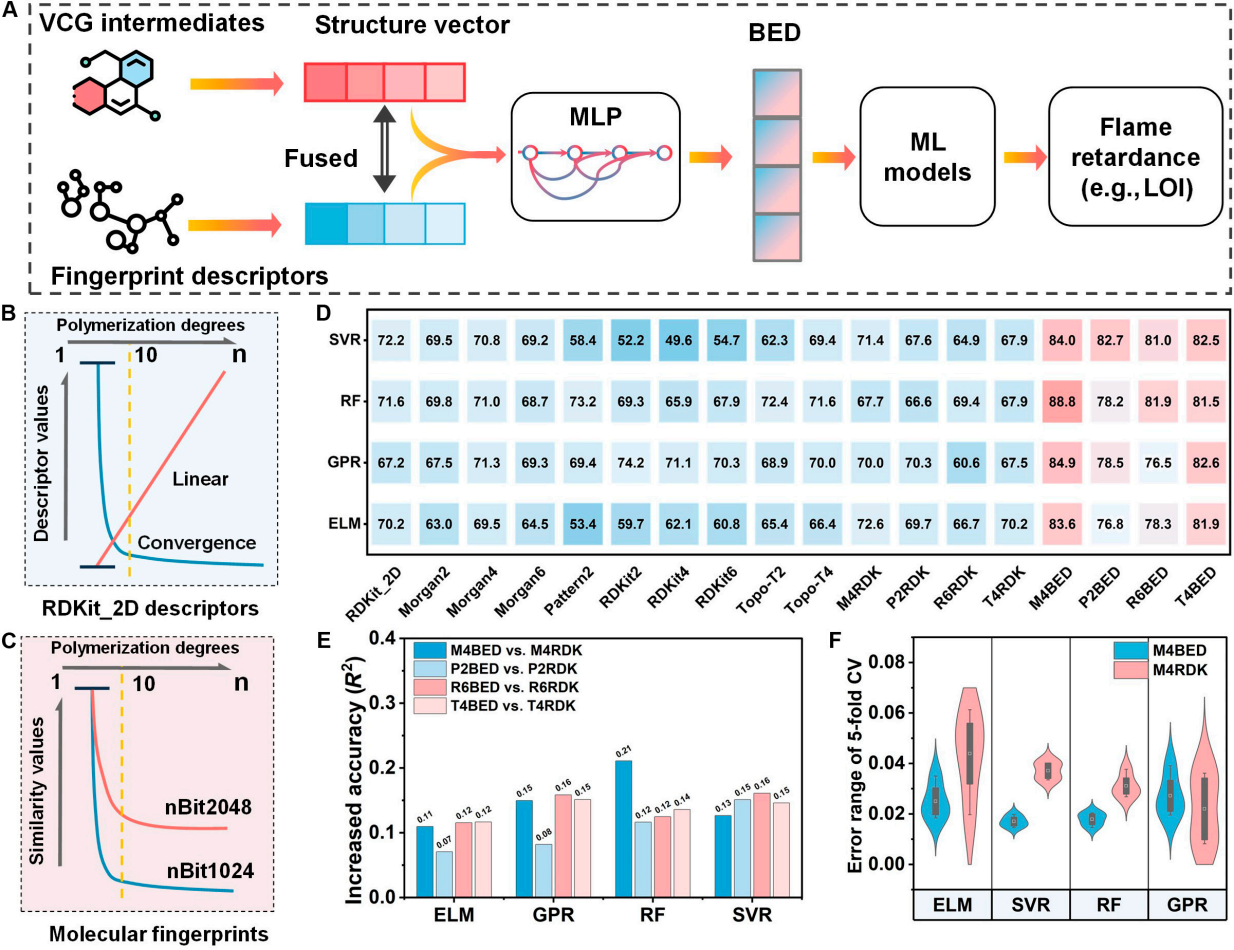
Feature construction and ML model validation based on BED. (A) The architecture of the BED construction and model prediction process. (B) The variation trend of RDKit_2D molecular descriptor values as the polymerization degree increases. (C) The variation in molecular fingerprint similarity between 1,024-bit and 2,048-bit fingerprints as the polymerization degree increases. (D) The matrix displays the verification accuracy (*R*^2^, %) of different descriptors within 4 machine learning models. (E) The increased accuracy of BED descriptors fused with intermediate information in 4 machine learning models. (F) The 5-fold cross-validation accuracy error in 4 ML models for M4BED and M4RDK.

Flame-retardant performance is not solely related to the structure sequence, and is more strongly related to the burning behavior. As shown in the training framework (Fig. 4A), we propose a feature processing method that embeds the massive burning intermediates extracted from VCG into polymer structure features. In the embedding approach, the VCG intermediates are converted into Morgan fingerprints to calculate the similarity from original structure, which are used for initial weights during embedding process. Inconsistent substructures (differential structures) in intermediates and original polymer structures are extracted into vectors for embedding. The fused feature, namely, burning embedding descriptors (M4BED, P2BED, R6BED, and T4BED), is further obtained through a multilayer perceptron (MLP) and input into the training processes, and the flame retardancy (e.g., limiting oxygen index [LOI], one of the most important indicators to characterize the flame retardancy) is output as labels. Extreme learning machine (ELM), random forest (RF), support vector machine regression (SVR), and Gaussian process regression (GPR) are deployed as the training models. In addition, models trained by traditional descriptors (RDKit_2D, Morgan2, Morgan4, Pattern2, RDKit2, RDKit4, RDKit6, Topo-T2, Topo-T4, M4RDK, R6RDK, and T4RDK, illustrating in the methods parts) are also executed as comparisons.

The training dataset (Fig. S6), directly determining the quality of the ML model, has recorded polymers (composed of C, H, O, N, S, P, F, Cl, and Br elements) and their flame retardancy was tested at the same burning conditions to ensure the consistency of the data. Data are from the literature and our lab work. The data distribution of flame retardancy is wide and uniform (the diversity of chemical composition and distribution are visualized in Figs. S6 and S7), indicating that these data exhibit representativeness, balance, and diversity in both chemical (Fig. S6E to G) and numerical aspects (Fig. S6B to D), making them suitable for constructing reliable training models.

To eliminate the effects of molecular weight on the performance, we compare RDKit_2D descriptor parameter changes along with the degree of polymerization. The curves of different descriptors that changed with the degree of polymerization are presented in the Supplementary Materials (Fig. S8). As shown in Fig. 4B, the value trends of the descriptors exhibit 2 types when the length of the polymer sequence structure reaches a certain level. One type of descriptor (such as BalabanJ, MinAbsEStateIndex, QED, etc.) that is related to molecular polar surface area, electronic topological index, or the photon polarization operator gradually converges with the increase of polymerization degree. Other calculated descriptors (such as Chi0n, SlogP_VSA, and Kappa1 to Kappa3) are based on pre-calculated surface area values of the electron cloud density distribution, increasing linearly with the appearance and growth of repetitive polymer degree.

Moreover, molecular fingerprint descriptors are extracted from different methods (Morgan, Pattern, RDKit, and Topological Torsion) to obtain richer chemical information of the polymer sequences, and the impact of polymerization degrees on molecular fingerprint descriptors is investigated in detail. Here, the Tanimoto similarity is compared between the molecular fingerprint vector (nBits = 1,024 or 2,048) with different polymerization degrees (1 to 66) in Fig. S9. The molecular fingerprint vector with 2,048 bits can extract more molecular structure information than that with 1,024 bits, and the similarity of the fingerprint vector with 2,048 bits, whose polymerization degree is 2 to 66, exhibits a lower value than that of the fingerprint vector with 1,024 bits. Comprehensively, in Fig. 4C, the similarity of fingerprint vectors gradually converges to a certain value, when polymerization degrees increase to over 10, indicating that there is nearly no longer obvious difference in the polymer structure captured by the fingerprint vectors. Hence, polymer chains, whose polymerization degree is 10, are optimally selected after balancing the trade-off among descriptor information, the reasonable calculation time, and computational complexity.

The proposed feature engineering strategy offers remarkable advantages. In the accuracy matrix (Fig. 4D, the detail accuracy sheet is given in Tables S3 and S4), before embedding the combustion intermediates, the low prediction accuracy is in the range of 49.6% to 74.2%, illustrating that the feature only extracting the polymer structure is insufficient for effectively training genomic models of flame-retardant properties, which is not only determined by the polymer sequence but also strongly related to the burning behavior. In contrast, our feature engineering strategy, incorporating combustion intermediates into structural descriptors, achieves high accuracy across various ML models. After embedding combustion intermediate vectors, the accuracy of all ML models exceeds 75.0% and shows the best result (88.8%). As shown in Fig. 4E, the growth of the accuracy increases by 7.1% to 21.1% after embedding the combustion intermediates, and the RF model training with the M4BED is confirmed as the optimal model. Additionally, in Fig. 4F, the trained models using M4BED exhibit lower error rates (0.025, 0.017, 0.018, and 0.027, respectively) than that of M4RDK (0.043, 0.037, 0.031, and 0.022, respectively), also indicating the better adaptability of the proposed feature engineering strategy.

Based on the above results, our feature engineering strategy serves as a more creative method for predicting the flame-retardant performance of applied polymeric materials by capturing the combustion feature of polymer sequence. The created reliable genomic model provides a basis for high-throughput screening materials with high flame-retardant performance.

### Polymer discovery and verification

**Fig. 5.**
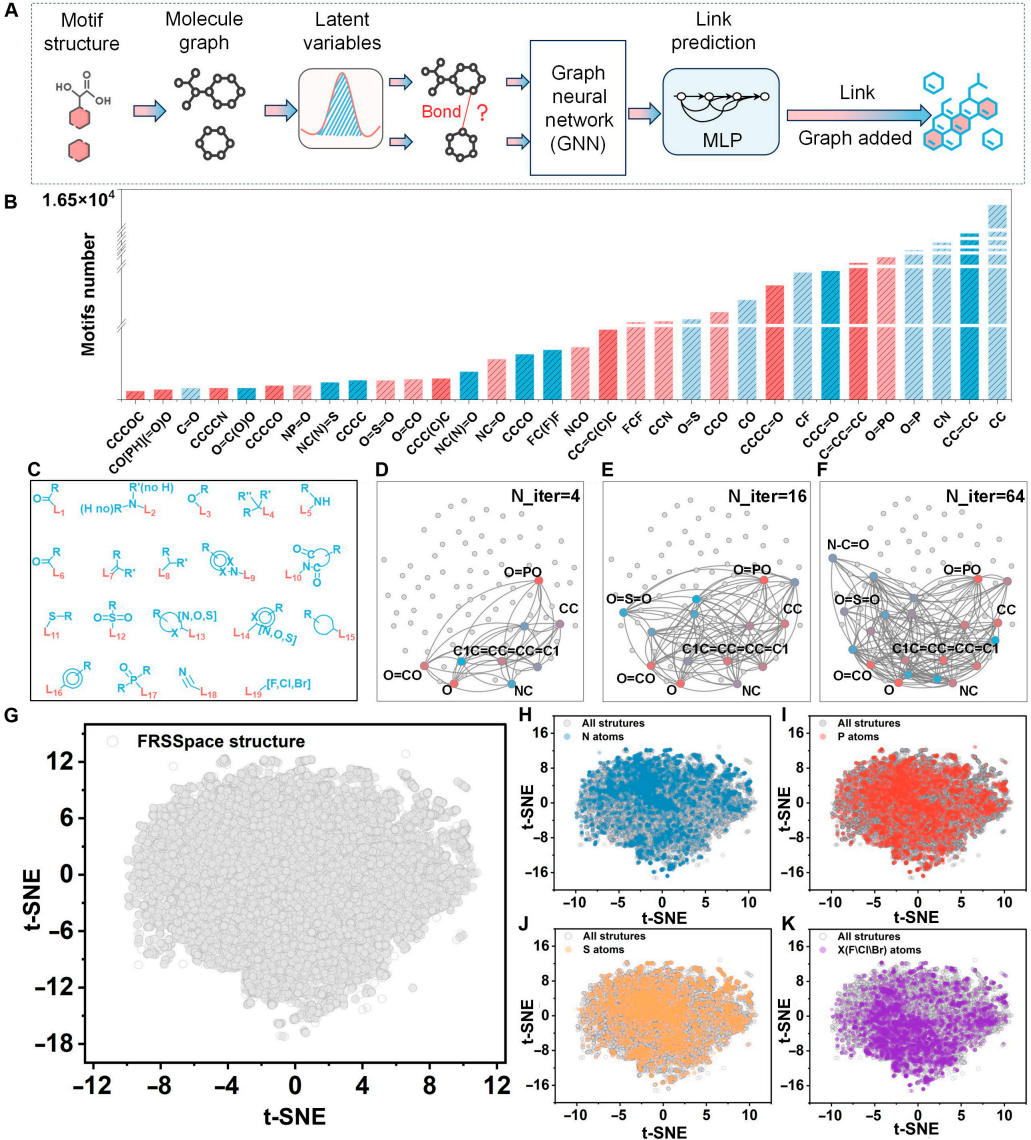
Visualization of structure space and experimental verification. (A) The assembly process of chemical space. (B) Statistics of motifs composed of different atom numbers. (C) Sets of fragments used to generate motifs. (D to F) Chemical reaction assembly networks with different iteration numbers. (G) Chemical space of flame-retardant structures. (H to K) Distribution of structures containing N, P, S, and F/Cl/Br elements in the chemical space.

The scientific discovery framework is practically applied further for accelerating polymer materials design. The search space is first constructed according to the flowchart in Fig. 5A. A molecular assembly task is based on a variational autoencoder to generate chemical spaces containing various structure units from scratch. Before searching the chemical space, we screen groups originating from large amounts of combustion intermediates from VCG. Different groups containing 2 to 5 atom counts generated by the VCG module are classically counted to effectively prefer motifs, as shown in Fig. 5B. There are 7 types of groups composed of 2 atoms, in which the C-C groups are the most abundant, exceeding 16,000 occurrences, the C-N groups are the second most abundant with around 660 occurrences, followed by the O=P groups with over 500 occurrences. We also calculate occurrences composed of 3 atoms. The O=PO, CCO, CCN, and NCO groups exhibit relatively high occurrences (415, 227, 208, and 109, respectively). The above statistical data suggest that incorporating fragments containing N or P elements will mainly unleash flame-retardant effects during burning processes; thus, it is a key approach toward inhibiting polymer burning. The structure containing 4 or 5 atoms are also given, in which cyclic or heterocyclic structures, such as C=CC=CC or NC(N)=O, are high frequency, suggesting that cyclic structures are also the main ingredient during burning processes.

Based on the aforementioned results and experimental experience, 19 sets of fragments (Table S5) are preferred and form 249 motifs (Table S6), shown in Fig. 5C. L_n_ represents the linking points of chemical bonds, and the number n is the identification code. R and R′ means variable functional groups. Figure 5D to F illustrates the visualized assembly networks with different iteration counts (N_iter = 4, 16, or 64), highlighting the formation process of phosphorus-containing structure units in color as an example. The whole chemical space (Fig. 5G, over 50k structures) assembled from motifs are searched containing H, C, N, O, F, P, S, Cl, and Br elements. Specific structures in the space containing P, N, S, or halogen elements are respectively marked in Fig. 5H to K. There are also some structures containing P and N at the same time, indicating the potential for discovering synergistic flame-retardant modification systems (some novel flame-retardant structures are listed in Table S8).

**Fig. 6.**
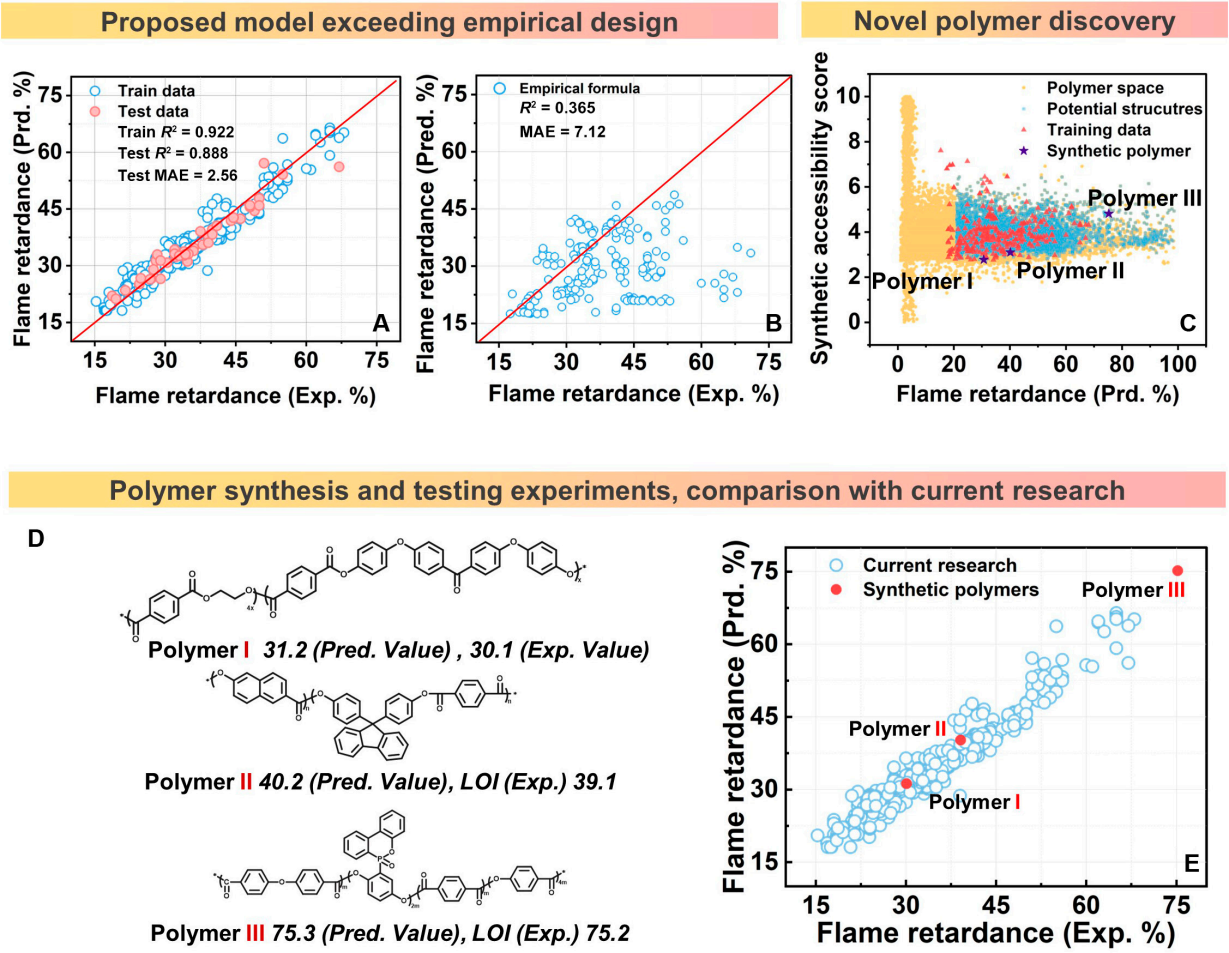
Screening novel polymeric materials with high flame retardancy. (A) Predicted data from genomic model vs. experimental data. (B) Predicted data from Van Kraven’s empirical model vs. experimental data. (C) Filtered novel polymers considering both the high flame retardancy and synthetic accessibility score. (D) The synthesis and testing experiments of 3 candidate polymers. (E) Comparison of novel discovery polymer with current research.

In discovering novel polymeric materials using the proposed genomic model, Fig. 6A illustrates the robust consistency between the experimental and predictive values, obviously exceeding the traditional empirical formula[43] that predicted data are almost completely wrong compared to experimental data (Fig. 6B). The mean absolute error (MAE) of the genomic model between the experimental and predicted values of the training dataset is only 2.56, far below that (7.12) of the empirical formula widely accepted in the research area. Furthermore, combining chemical space search and performance prediction based on the genomic model here exhibits great potential for rapidly discovering novel materials. In Fig. 6C, 4,943 types of polymers are preferred from the entire chemical space according to the following filter rules (LOI ≥ 20.95 and synthetic accessibility score ≥ 2.00). Boundary conditions are confirmed from expert experience and the current work (LOI of existing polymers being generally in the 17.3 to 68.0 range, and synthetic accessibility score being in the 2.765 to 7.615 range). Three novel polymers (Polymer I, Polymer II, and Polymer III) are also synthesized, and their flame retardancy is tested in Table S7 to verify the feasibility of the proposed discovery framework. Figure 6D illustrates the high consistency of the experimental data (Polymer I, 30.1; Polymer II, 39.1; Polymer III, 75.2) and predicted data (Polymer I, 31.2; Polymer II, 40.5; Polymer III, 75.3). Significantly, Polymer III exhibits a quite high flame retardancy surpassing previous published work as shown in Fig. 6E. Results indicate that the feature engineering strategy fused both polymer structure and combustion intermediates, achieving the generalizable model with exceptional universality and accuracy, thus serving as an efficacious toolkit for the rapid exploration and predictive estimation of flame retardancy.

## Discussion

This work demonstrates a discovery framework that can effectively explore novel polymers with high flame-retardant performance. The created PBOA overcomes limitations of traditional methods that often require a variety of tests and only obtain very few combustion characteristic parameters, enabling synchronized analysis of massive data in just a one-time test. It also exhibits wide applicability to analyze materials requiring thermal, fire, or ablation-resistant properties in harsh environments like aerospace, lithium batteries, outer space, etc. The VCG provides an attractive toolkit for extensively producing dozens of times more intermediate information on burning processes than traditional experimental methods. It can also parse different materials with the expansion and update of the reaction rules. A trained genomic model enables high accuracy for predicting the flame-retardant performance. The integration of multiple analytical techniques replaces some of the traditional experiments and tests, substantially reducing the time for discovering polymers with excellent flame retardancy. As accumulation for more burning reactions and intermediates from the created in situ burning analyzer, the benefits of using our proposed integrated toolkits for guidance will exponentially expand. In the future, the more iterations there are, the more accurate is the discovery workflow. In the future, the proposed paradigm may also inspire the discovery of novel materials in other fields such as thermal catalysis, sustainable materials, or energy materials.

## Methods

### Polymer burning operando analyzer

The in situ burning analysis process has the following 3 steps: real burning conditions, sampling and measurement, and data analysis.

Real burning conditions. The real burning conditions of materials are generated by 2 modes. One is to put the sample (100 × 100 × 4 mm^3^) on a load cell for further irradiation to a given uniform heat flux (in general from 10 to 100 kW/m^2^) produced by a conical radiant electrical heater from above. Further, the sample is ignited by an electric spark. The other is to let certain size samples (120 × 6.5 × 3.2 mm^3^) set in a quartz column that allows the burning of the specimen to be observed and in the specific atmosphere with different oxygen concentration; meanwhile, using a butane flame ignites the upper end of the specimen, and the subsequent burning behavior is monitored.

Sampling and measurement. The burning properties and the massive burning intermediate are synchronously sampled. The physical signals, such as the mass loss of the sample, the heater power, the heat temperature, and the testing time are acquired by PLC and communicated to the upper computer via an RS-232 to RS-422/RS-485 converter. The massive burning intermediates are sampled by a heated quartz needle using a nonintrusive optical approach. The heated quartz needle is directly inserted into the burning flame, and its rear end is connected to a 350°C heating tube (1/8 inch) with a quartz wool filter. The burning intermediates are passed through different analyzers, firstly tested by the nonintrusive optical method [44] (Fourier Transform Infrared spectrometer, FTIR), and subsequently entered the VUV photoionization time-of-flight mass spectrometer (VUV-TOF-MS) [45] (photon energy, 10.6 eV; resolution >2,000; detection limit >1 ppb; membrane sampling temperature, 250°C). The end of the sampling is connected to a high-temperature pump to provide negative pressure power for the entire sampling. The nonintrusive miniature spectrometer (wavelength range, 200 to 800 nm; slit, 10 μm; resolving resolution <1 nm) also directly collects the light signals of the flaming combustion. The oxygen, carbon monoxide, and carbon dioxide concentration are also analyzed according to the absorption spectroscopy principle and Beer–Lambert law. Before the sampling, the complex gas is filtered by the quartz wool. The measurement synchronously captures wavenumbers, Mass-to-Charge Ratio, wavelength of emission spectra, and gas concentration data at the same moment. Meanwhile, a homemade smoke sampling module (SSM) is constructed to collect smoke soot generated by the burning of materials. The cores of this device are the smoke holder and the filter cartridge. The inner diameter of the smoke holder needs to be changed according to the flow rate of the soot in the pipeline, which makes sure that the mature soot can be inhaled in the filter cartridge. Moreover, the working conditions such as the suction force of the pump can be adjusted through the setter. In addition, since the sample acquisition time is different for each device, we align the acquisition moments by calculating the gas flow rate.

Data analysis. According to the wavenumbers, mass-to-charge ratio, wavelength of emission spectra, and gas concentration parameters at the same moment, the chemical bonds, exact chemical structure, and diatomic free radical information of intermediates are confirmed. These data are also updated to fill the *PolymData* (https://polymdata.scu.edu.cn).

### Virtual combustion generator

The mapping approach from the PBOA to VCG is discussed in the following. Burning intermediates of polymer burning are collected based on the PBOA analysis data combined with the reference results (from thermogravimetric analysis–mass spectrometry, thermogravimetric analysis–Fourier transform infrared, and pyrolysis–gas chromatography–mass spectrometry), also along with the expert experience to enhance the reliability.

We converted the collected intermediates’ information output into simplified molecular input line entry specification (SMILES) [46,47] expressions as the chemical structure template. When determining reactant expressions, the reaction core is defined by determining which product atoms have different connectivity from the corresponding reactant atoms; then, it is expanded to include adjacent unmapped leaving groups and immediately neighboring atoms. Neighboring atoms are fully generalized into any non-hydrogen substituent for maximal generality to achieve high coverage at the expense of low specificity. A SMIRKS [48] string encoding the substructure pattern at the reaction core was generated for the reactants and for the products, which together are defined as reaction SMIRKS strings. A total of 44 unique reaction SMIRKS strings are extracted from different polymers. In addition to the marked product templates, other parts that do not belong to the core of the SMIRKS reaction will generate free products, which also serve as fuels during the burning reaction and gradually cleave into active small fragments, which cannot be captured by realistic tests.

The t-distributed stochastic neighbor embedding (t-SNE) algorithm is utilized to reduce the chemical distribution of the obtained VCG products to a 2-dimensional space.

### Machine learning genomic models

#### Dataset

The dataset comprises 1,624 sets of performance data for various (230) polymer systems, by leveraging application architectures (Elsevier and Springer Nature APIs) accessing literature webpages, along with a manual collection of burning properties and their relational parameters from the reference and our lab data, including LOI, char residue (CR%), glass transition temperature (*T*_g_), initial decomposition temperature (*T*_5%_), maximum decomposition temperature (*T*_max_), melting temperature (*T*_m_), heat release, and smoke release.

#### Structure descriptors

All polymerization degrees were converted into SMILES, which were generated into computable objects by the RDKit MolFromSmiles package.

RDKit_2D descriptors, totally containing 88 descriptors (Fig. S8), are extracted by Python RDKit 2021.09.1 [49] package.

Morgan fingerprints (also called extended-connectivity fingerprints) are implemented by the Python RDKit package, the calculation diameters are 2, 4, and 6, respectively, marked as Morgan2, Morgan4, and Morgan6. RDKit fingerprints are calculated with the atomic diameters used in the calculation being 2 (RDKit2), 4 (RDKit4), and 6 (RDKit6). Parameters in the RDKit package are as follows: nBits = 1,024 or 2,048, minPath = 1, maxPath = 7, and useHs = True. Topological torsions are calculated by targetSize = 2 and 4, nBits = 1,024 or 2,048, which are represented as Topo-T2 and Topo-T4, respectively. The calculation diameter for Pattern fingerprints is 2, while the fpSize is 1,024 or 2,048, marked as Pattern2. We utilize the aforementioned 9 molecular fingerprints as features for inputting into 4 ML models, and the resulting test accuracy (*R*^2^) is provided in Table S3.

The RDK descriptors are derived from the fusion of RDKit_2D descriptors and 4 types of molecular fingerprints. Based on the accuracy data in Table S3, we chose 4 types of molecular fingerprint calculation methods (Morgan4, Pattern2, RDKit4, and Topo-Torsions2) to generate 4 molecular fingerprint vectors with a length of nBit = 1,024/2,048. To mitigate errors caused by the large range of RDKit_2D descriptors, we first normalize the RDKit_2D descriptors and molecular fingerprint vectors, then merge them using the concat method to create descriptors. Following the accuracy results in Table S4, we ultimately selected 4 fused RDK descriptors: M4RDK, P2RDK, R6RDK, and T4RDK.

#### Burning embedding descriptors

The construction process of the BED descriptors is detailed as follows: Taking M4BED as an example, the intermediates and original sequences outputted by VCG are initially converted into Mol objects using RDKit. A 2,048-bit Morgan fingerprint is then calculated with a radius of 4, and the Tanimoto similarity between these intermediates and sequences is computed and recorded. Subsequently, the intermediates and original structures from VCG are transformed into Molgraph, and inconsistent structures are extracted through atom comparison in the adjacency matrix. Following this, all intermediates derived from a polymer sequence undergo encoding by variational autoencoder (VAE) to produce a latent space vector of length 256. The VAE encoding section comprises 2 fully connected hidden layers utilizing rectified linear unit (ReLU) as the activation function, eventually outputting a 256-bit latent space mean vector. After reconstructing the intermediate information outputted by VCG using VAE, a 3-layer MLP is employed to linearly transform both the VCG information and the 4 molecular fingerprints (nBit = 2,048). This process results in the generation of the burning embedding descriptor M4BED.

Similarly, descriptors P2BED, R6BED, and T4BED are obtained through identical construction processes for calculating the respective molecular fingerprint information. The accuracy of all models is displayed in Table S4.


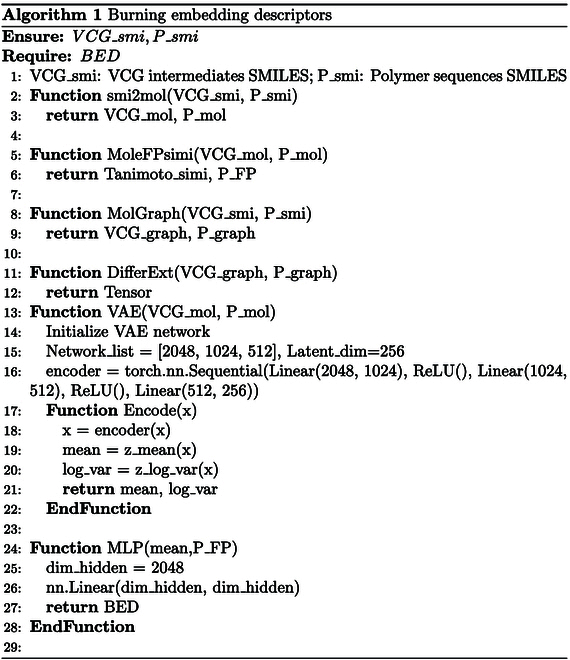


#### Regressor

In this section, the dataset is randomly divided into 5 parts, including 184 training data and 46 testing data. The LOI was used as burning property labels, and all evaluation indicators were evaluated by 5-fold cross-validation.

We evaluated the performance of the BED using 4 different machine learning techniques: ELM, RF, SVR, and GPR.

The ELM was designed as a single-layer feedforward neural network. The model contains a random selection of input weights and hidden nodes. During training, the input weights are used to calculate the outputs of the hidden nodes, which are then passed through an activation function to determine the predictions.

The SVR model is a type of machine learning algorithm that can be used for regression problems. The initial kernel parameters for the SVR model include a radial basis function (RBF) kernel, a polynomial kernel degree of 3, kernel scaling parameter of scale, a tolerance parameter of 0.1, a regularization parameter of 1, and an epsilon parameter of 0.1. The model includes a decision tree as the estimator, with 100 nodes, using a “squared_error” feature selection standard and minimizing L2 loss using the average value for each terminal node. The minimum number of samples required per leaf node is 1, and the minimum number of samples required to split an internal node is 2.

The RF model is another type of machine learning algorithm used for regression problems. The model’s initial parameters include a decision tree estimator with 100 nodes, using a “squared_error” feature selection standard and minimizing L2 loss using the average value for each terminal node. The minimum number of samples required per leaf node is 1, and the minimum number of samples required to split an internal node is 2.

The GPR model is a type of machine learning algorithm used for regression problems. The initial kernel parameters include a ConstantKernel * RBF alpha parameter of 1 and an optimization algorithm of “fmin_l_bfgs_b”. A brief description with additional details is provided in the Supplementary Materials.

Before training the model, the dataset was randomly split into a training dataset (80%) and a test dataset (20%). The following metrics were used to evaluate the quality of the model:

*R*^2^ measures the proportion of variance in the dependent variable that is explained by the independent variables. The formula for this metric is as follows:

$$\begin{array}{c} \;R^{2}=1-\frac{\text{sum}\;\text{squared}\;\text{regression}\;(\text{SSR})}{\text{total}\;\text{sum}\;\text{of}\;\text{squares}\;(\text{SST})}\\ =1-\frac{\sum_{i=1}^{n}{\left(\hat{y_{i}}-y_{i}\right)}^{2}}{\sum_{i=1}^{n}{\left(\bar{y_{i}}-y_{i}\right)}^{2}} \end{array}$$ (1)

MAE indicates the mean of the absolute errors between predicted and true values as shown in the following:

$$\text{MAE}=\frac{1}{n}\sum_{i=1}^{n}\left|y_{pre,i}-y_{\text{ture},i}\right|\;$$ (2)

To further ensure the quality of the LOI model after optimization, all evaluation metrics were subjected to 5-fold cross-validation to obtain the average value with 1 to 200 iterations.

### Polymer discovery and verification

#### Assembled chemical space

We referred to the BRICS rules implemented in RDKit and combined with the burning characteristics of polymers to define 19 atomic environments (Fig. 6C). Subsequently, the VCG model-generated heat decomposition mid-products were dissected into the smallest motifs.

The fragment set generated can be viewed as a molecular graph with disconnected subgraphs, lacking bonds between them. We formalize the completion of these bonds as a link prediction task, which is a familiar concept to the GNN community. To achieve this, we first use graph neural networks to encode molecular graphs into latent variables. For each latent variable, the iteration number “max_iter” was set to optimize the generation of latent variables in order to more effectively represent the structural and flame-retardant features of the motif (the optimization and linking prediction of the latent variables are visualized in Fig. 5D to F using the N_iter = 4, 16, or 64 iteration number). Then, given node u and v in 2 different latent variables, we use a 3-layer MLP with ReLU activation to predict whether the bond exist or not.

The predicted edges are sorted in descending order based on the bond prediction result and then added to the graph turn if the bond confidence level is higher than 0.5 by randomly selecting the connectivity fragments. Valence and ring checks are performed during the connection process in the next fragment to reject connections that violate valence or form unstable rings that are either too small or too large. When outputting the fragment, duplicates with identical sequences are dropped to ensure the uniqueness of the structure in space.

Furthermore, the assembled chemical space is compressed into a 2-dimensional format using the t-SNE algorithm.


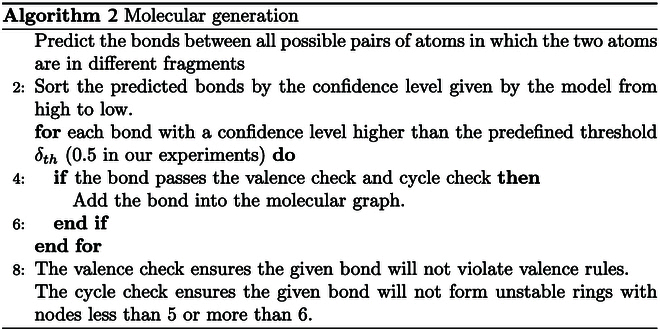


#### Screening candidate structures from chemical space

Step 1: Convert the structures in the *PolymData* dataset and FRSSpace to Mol objects using the RDKit package.

Step 2: Calculate the SAScore of polymers in the *PolymData* dataset; the SAScore range is 2 to 6.

Step 3: Calculate the SAScore of all structures in FRSSpace and mark the structures with calculated values of 2 to 6 as “Synthetic”.

Step 4: Calculate the flame retardancy of all structures in FRSSpace using the M4BED-based RF model.

Step 5: Screening the candidate structures that satisfy the flame retardancy performance greater than 20.95 from the structures labeled as Synthetic.

#### Empirical formula

Van Krevelen (1974) found an empirical formula using the correlation between LOI and char residue (CR) on pyrolysis: $\mathrm{LOI}=\frac{17.5+0.4\mathrm{CR}}{100}$, where CR is the carbon residue measured by thermogravimetric analysis.

#### Polymer discovery

The synthetic accessibility score is quantified by calculating molecular fingerprints and fragment scores of molecules, molecular size (MW, atom number), stereochemistry, spirocycles, and bridge features, and mapping the scores of these features to the 1 to 10 interval.

The synthesis process of the target polymer mainly uses several compounds {Polymer I: bis(4-(4-hydroxyphenoxy)phenyl)methanone, 1,3-dioxolan-2-one, and dimethyl terephthalate; Polymer II: 6-hydroxy-2-naphthoic acid, terephthalic acid, and 4,4′-(9H-fluorene-9,9-diyl)diphenol; and Polymer III: 4-(propionyloxy)benzoic acid, terephthalic acid, and 2-(6-oxidodibenzo[c,e][1,2]oxaphosphinin-6-yl)-1,4-phenylene diacetate, 4,4′-oxydibenzoic acid} and is obtained through esterification/acylation and melt polycondensation processes. During the reaction process, esterification and polycondensation catalysts are added. The entire synthesis process is carried out in a 3-necked flask. The esterification stage is in the nitrogen atmosphere, and the reaction temperature is 190 to 260 °C. The polycondensation process is carried out in a high-temperature and high-vacuum environment, and the reaction temperature is 260 to 330 °C.

## Data Availability

Supplementary Materials, containing details of the theoretical aspects, machine learning methodology, and experimental measurements, are available for this paper. The featurization, prediction, and trained data are available from https://github.com/adenkics/FBRPolyInfo/tree/main/FirPlotfm.
