# Supplementary material for: Scientific Discovery Framework Accelerating Advanced Polymeric Materials Design
Source: Research (Wash D C). 2024 Jul 8;7:0406. doi: 10.34133/research.0406 (PMC11228074; doi:10.34133/research.0406)
Supplement: Supplementary 1 — Figs. S1 to S9 Tables S1 to S8 [file research.0406.f1.zip › Supplemental Material.docx]

**Supplementary Information**

**Scientific Discovery Framework Accelerating Advanced Polymeric Materials Design**

*Ran Wang, Teng Fu*, Ya-Jie Yang, Xuan Song, Xiu-Li Wang, Yu-Zhong Wang^*^*

The Collaborative Innovation Center for Eco-Friendly and Fire-Safety Polymeric Materials (MoE), National Engineering Laboratory of Eco-Friendly Polymeric Materials (Sichuan), State Key Laboratory of Polymer Materials Engineering, College of Chemistry, Sichuan University, Chengdu, 610064, China.

*Corresponding author. Email: Teng Fu (Futeng@scu.edu.cn) and Yu-Zhong Wang (yzwang@scu.edu.cn)

# Supplementary Materials


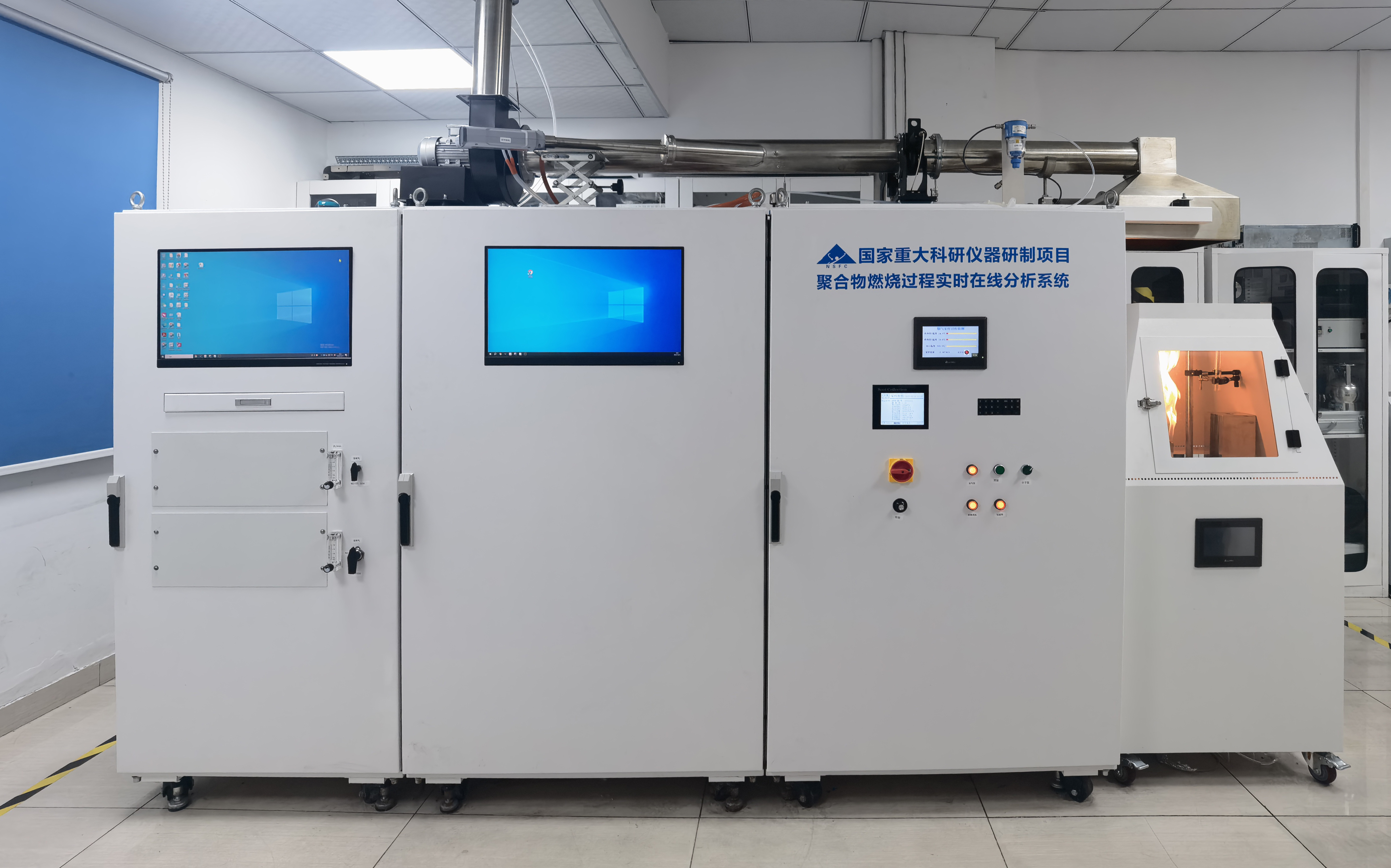


Figure S1.

Polymer burning operando analyzer

Figure S2.

Polymer Spectral Database (Polymdata, access URL: polymdata.scu.edu.cn), includes the IUPAC names, SMILES expressions, molecular formulas, molecular weights, exact masses, and m/z values (calculated for the singly charged positive ions, using H+ as an example) of the generated structures, as well as their 2D structure diagrams.

Figure S3.

Burning parameters obtained synchronously by PBOA analyzer.

(A) The measurement time range was set to 1-500 seconds, and mass spectra of combustion products were collected at 20 seconds, 150 seconds, and 210 seconds.

(B) Infrared spectra of burning products were obtained through temperature-programmed measurements controlled by PBOA.

(C) Spectral data revealing the presence of free radicals released within the combustion flame were captured.

(D) and (E) respectively provide Raman spectrum data and XPS electron spectrum data for soot produced during the combustion of EP materials added with flame retardants.

(F) PBOA-TDLAS was employed to monitor changes in the concentrations of oxygen, carbon monoxide, and carbon dioxide throughout the 0-500 second combustion period.

Figure S4.

Collected reaction rules for semi-polyesters, PET copolymers containing benzamide structures undergo a special thermal decomposition reaction.

Figure S5.

Detailed SMIRKS reaction expressions for resolving polymer sequence combustion products in the VCG module.

Figure S6.

(A), The scatterplot of 1624 points from the dataset draft the data contribution and indicate the high correlation among limit oxygen index (LOI, in the range of 25.0~75.0), the glass transition temperature (Tg, in the range of 0 to 421°C), the melting temperature (Tm, in the range of 49.0~405.4°C), the char ability (CR, in the range of 19.5.0~73.0), the thermal stability (T5%, in the range of 128.0~573.0), the temperature at the max decomposition rata (Tmax, in the range of 219.0~682.0).

(B), (C), and (D) respectively illustrate the correlation and regression relationships between the flame retardancy properties of Limiting Oxygen Index (LOI), Char Residual (CR), and Glass Transition Temperature (Tg).

(E), (F), and (G) represent the chemical space distribution of polymers with tested performance values of LOI, CR, and Tg, respectively. Principal Component Analysis generated two principal components, PC1 and PC2, which are assigned to the axes in the Figure For each polymer, the color variation represents the proportion of sp3-hybridized carbon atoms, the shape of the data points corresponds to different ranges of normalized TPSA values, and the size of the data points indicates the number of rings present in the polymer structure.

Figure S7.

Training dataset for ML model, in addition to the fundamental C/H/O elements, the polymer compositions encompass elements such as N, P, S, and halogens. (a), (b), (c), and (d) depict chemical spatial representations of polymer sequences featuring atoms of S, P, N, and X (F/Cl/Br), respectively. The x-axis represents the Limiting Oxygen Index (LOI) of polymers in the dataset, while the y-axis represents the total degree number of rings in degree of polymerization. The z-axis TPSA parameter represents the cumulative surface area of polar molecules within polymerization degree sequences. Colored regions denote the distribution of structures containing S, P, N, and X (F/Cl/Br) atoms, while the grey region represents the distribution across the entire dataset.

Figure S8.

Factors influencing the accuracy of polymer structural feature information and commonly used descriptors in different ML models. One class of descriptor values gradually converges with increasing degree of polymer (red data), while the other class of descriptor values gradually increases (teal data). All values are calculated using the RDKit MolecularDescriptorCalculator package.

Figure S9.

(a), four molecular fingerprints with nBits=1024 (Morgan\Pattern\RDKit\Topological-Torisons) were calculated for different polymerization degrees structural information, respectively compared with the molecular fingerprint calculated with unit=1 to calculate Tanimoto similarity, as well as Tanimoto similarity of molecular fingerprints with nBit=2048(b).

Table S1.

key challenges for extreme-condition-sampling and data-synchronization solved by PBOA.

| Key challenges |
| --- |
| Stable work and sampling under high temperature flame  Instrument calibration, the gas flow rate and temperature detection module ensures the stability of the instrument during the test.  Noisy sampling intervals  Synchronize data from various sensors or sources in real time |

Table S2.

Bisphenol A polycarbonate burning intermediates generated by VCG (partable), the detailed data is in the excel sheet "Table S1 VCG intermediate(Bisphenol A polycarbonate)".

| PET burning intermediates SMILES |
| --- |
| O=C=O  c1ccccc1  cccccC=O  oc1ccccc1  CCc1ccccc1  c1ccc(O)cc1  O=Cc1ccccc1  C=Cc1ccccc1  [c+]1ccccc1  O=cc1ccccc1o  Oc1ccc(O)cc1  O=COc1ccccc1  CC=Cc1ccccc1  O[c+]1ccccc1  CCc1ccc(O)cc1  CC(C)c1ccccc1  O=C=Oc1ccccc1  … |

Table S3.

R^2^ values predicted by four types of molecular fingerprints with different parameters as structural feature inputs for ML models.

| ML Model accuracy (R^2^) | | | | | | | | |
| --- | --- | --- | --- | --- | --- | --- | --- | --- |
| Descriptors^a^ | nBits=1024 | | | | nBits=2048 | | | |
|  | ELM | GPR | RF | SVR | ELM | GPR | RF | SVR |
| Morgan2 | 0.630 | 0.675 | 0.698 | 0.695 | 0.584 | 0.680 | 0.712 | 0.714 |
| Morgan4 | 0.683 | 0.693 | 0.710 | 0.703 | 0.701 | 0.702 | 0.704 | 0.719 |
| Morgan6 | 0.645 | 0.693 | 0.687 | 0.692 | 0.609 | 0.663 | 0.728 | 0.712 |
| Pattern2 | 0.696 | 0.676 | 0.708 | 0.670 | 0.678 | 0.685 | 0.682 | 0.667 |
| RDKit2 | 0.597 | 0.722 | 0.693 | 0.522 | 0.628 | 0.708 | 0.705 | 0.471 |
| RDKit4 | 0.621 | 0.711 | 0.659 | 0.496 | 0.613 | 0.715 | 0.674 | 0.492 |
| RDKit6 | 0.654 | 0.611 | 0.677 | 0.676 | 0.643 | 0.665 | 0.689 | 0.660 |
| Topo-Torsions2 | 0.654 | 0.689 | 0.724 | 0.623 | 0.600 | 0.675 | 0.698 | 0.645 |
| Topo-Torsions4 | 0.618 | 0.558 | 0.620 | 0.629 | 0.639 | 0.626 | 0.655 | 0.645 |

a, Morgan fingerprint (also called as Extended-connectivity fingerprints ) are implemented by Python RDKit package, the calculation diameters are 2, 4, and 6, respectively, marked as Morgan2, Morgan4, Morgan6. RDKit fingerprint are calculated with the atomic diameters used in the calculation being 2 (RDKit2), 4 (RDKit4), and 6 (RDKit6). Parameters in the RDKit package are as following: nBits=1024 or 2048, minPath=1, maxPath=7, useHs = True. Topological torsions are calculated by targetSize=2 and 4, nBits = 1024 or 2048, which are represented as Topo-torsion2 and Topo-torsion4 respectively. Pattern fingerprints use fpSize = 1024 or 2048, others were default calculation parameters.

Table S4.

R^2^ values of fused molecular fingerprint and burning embedding descriptors calculated with different bits as structural feature inputs for ML models.

| ML Model accuracy (R^2^) | | | | |
| --- | --- | --- | --- | --- |
| Descriptors^a^ | ELM | GPR | RF | SVR |
| RDKit_2D | 0.702 | 0.672 | 0.716 | 0.722 |
| M4RDK(1024) | 0.731 | 0.680 | 0.687 | 0.701 |
| M4RDK(2048) | 0.726 | 0.700 | 0.677 | 0.714 |
| P2RDK(1024) | 0.722 | 0.689 | 0.653 | 0.686 |
| P2RDK(2048) | 0.697 | 0.703 | 0.666 | 0.676 |
| R6RDK(1024) | 0.682 | 0.629 | 0.684 | 0.650 |
| R6RDK(2048) | 0.667 | 0.606 | 0.694 | 0.649 |
| T4RDK(1024) | 0.725 | 0.656 | 0.680 | 0.704 |
| T4RDK(2048) | 0.702 | 0.675 | 0.679 | 0.679 |
| M4BED(1024) | 0.773 | 0.793 | 0.800 | 0.813 |
| M4BED(2048) | 0.836 | 0.849 | 0.888 | 0.840 |
| P2BED(1024) | 0.746 | 0.826 | 0.798 | 0.790 |
| P2BED(2048) | 0.768 | 0.785 | 0.782 | 0.827 |
| R6BED(1024) | 0.754 | 0.781 | 0.797 | 0.816 |
| R6BED(2048) | 0.783 | 0.765 | 0.819 | 0.810 |
| T4BED(1024) | 0.818 | 0.808 | 0.800 | 0.789 |
| T4BED(2048) | 0.819 | 0.826 | 0.815 | 0.825 |

a, M4RDK: Morgan fingerprint (radius = 4) + RDKit 2D descriptors; P2RDK: Pattern fingerprint (radius = 2) + RDKit 2D descriptors; R6RDK: RDKit fingerprint (maxPath = 6) + RDKit 2D descriptors; T4RDK: Topological Torsion fingerprint (targetSize=4) + RDKit 2D descriptors; M4BED, P2BED, R6BED, T4BED are M4RDK, P2RDK, R6RDK, T4RDK embedded with burning intermediates outputted by VCG.

Table S5.

BRICS-based primitive acquisition rules.

| SMARTS expression |
| --- |
| L1: [C;D3]([#0#6#7#8])(=O)  L2: [N;!R;!D1;!$(N=*)]-;!@[#0#6]  L3: [O;D2]-;!@[#0#6#1]  L4: [C;!D1;!$(C=*)]-;!@[#6]  L5: [N;!D1;!$(N=*);!$(N-[!#6;!#16;!#0;!#1]);!$([N;R]@[C;R]=O)]  L6: [C;D3;!R](=O)-;!@[#0#6#7#8]  L7: [C;D2D3]-[#6]  L8: [C;!R;!D1;!$(C!-*)]  L9: [n;+0;$(n(:[cnos]):[cnos])]  L10: [N;R;$(N(@C(=O))@[CNOS])]  L11: [S;D2](-;!@[#0#6])  L12: [S;D4]([#6#0])(=O)(=O)  L13: [C;$(C(-;@[CNOS])-;@[NOS])]  L14: [c;$(c(:[cnos]):[nos])]  L15: [C;$(C(-;@C)-;@C)]  L16: [c;$(c(:c):c)]  L17: [$(P(=[OX1])[#0#6]([#6])([#6]))]  L18: [C;D2]#[N;D1]  L19: [FClBrI][#6] |

Table S6.

249 motifs optimized by 19 sets of fragment rules, the detailed data in the excel sheet "Table S5 Motifs".

| 249 motifs |
| --- |
| [16*]c1cccc(N[Rb])c1  [16*]c1ccc(N)c(O)c1  [16*]c1ccc(O)cc1[16*]  [16*]c1cccc([Rb])c1  [16*]c1ccc(N=C)cc1  [16*]c1cccc([16*])c1O  [16*]c1cccc(O)c1  [16*]c1ccc2nc[nH]c2c1  [16*]c1ccccc1[Rb]  [16*]c1ccccc1O  [16*]c1cccc(C)c1  [10*]N1C=NCC1=O  [10*]N1CC([15*])CC1=O  [10*]N1CCC([15*])C1=O  [3*]OP([17*])(=O)[Rb]  [3*]O[PH](=O)O[3*]  [3*]O[Fr]  [3*]O[3*]  [3*]OP(=O)([Fr])O[3*]  [3*]OC=NC(C)C=O  [3*]ONCCCCC  [3*]O[Rb]  [3*]OC  [3*]OP(=O)([Rb])O[3*]  [3*]OP(=O)(O)O[3*]  [3*]ONCCCCC[8*]  … |

Table S7.

Thermal and fire properties of synthetic polymers.

| Sample | T_5%_^a^  (°C) | T_max_^a^  (°C) | CR ^a^  (wt%) | UL-94 ^b^ | LOI ^c^  (%) |
| --- | --- | --- | --- | --- | --- |
| Polymer I | 394 | 438 | 27.0 | V-2 | 30.1 |
| Polymer II | 497 | 531 | 61.5 | V-0 | 39.1 |
| Polymer III | 464.6 | 498.6 | 51.1 | V-0 | 75.2 |

^a^ TGA: heating rate of 10 °C/min from 40 to 700 °C under N_2_ atmosphere. ^b^ Vertical burning test was performed using a CZF-3 vertical burning test instrument with a sample size of 130 × 13 × 3.2 mm^3^. ^c^ Limiting oxygen index value were performed using an JF-3 oxygen index measurement according to ASTM D 2863 with sample size of 130 × 6.5 × 3.2 m

Table S8.

Flame retardant properties of candidate polymers containing ester groups (Pred.)

| ID | Structure |
| --- | --- |
| 1 |  |
| 2 |  |
| 3 |  |
| 4 |  |
| 5 |  |
| 6 |  |
| 7 |  |
| 8 |  |
| 9 |  |
| 10 |  |
