# Supplementary figures and images for: Scientific Discovery Framework Accelerating Advanced Polymeric Materials Design
Source: Research (Wash D C). 2024 Jul 8;7:0406. doi: 10.34133/research.0406 (PMC11228074; doi:10.34133/research.0406)

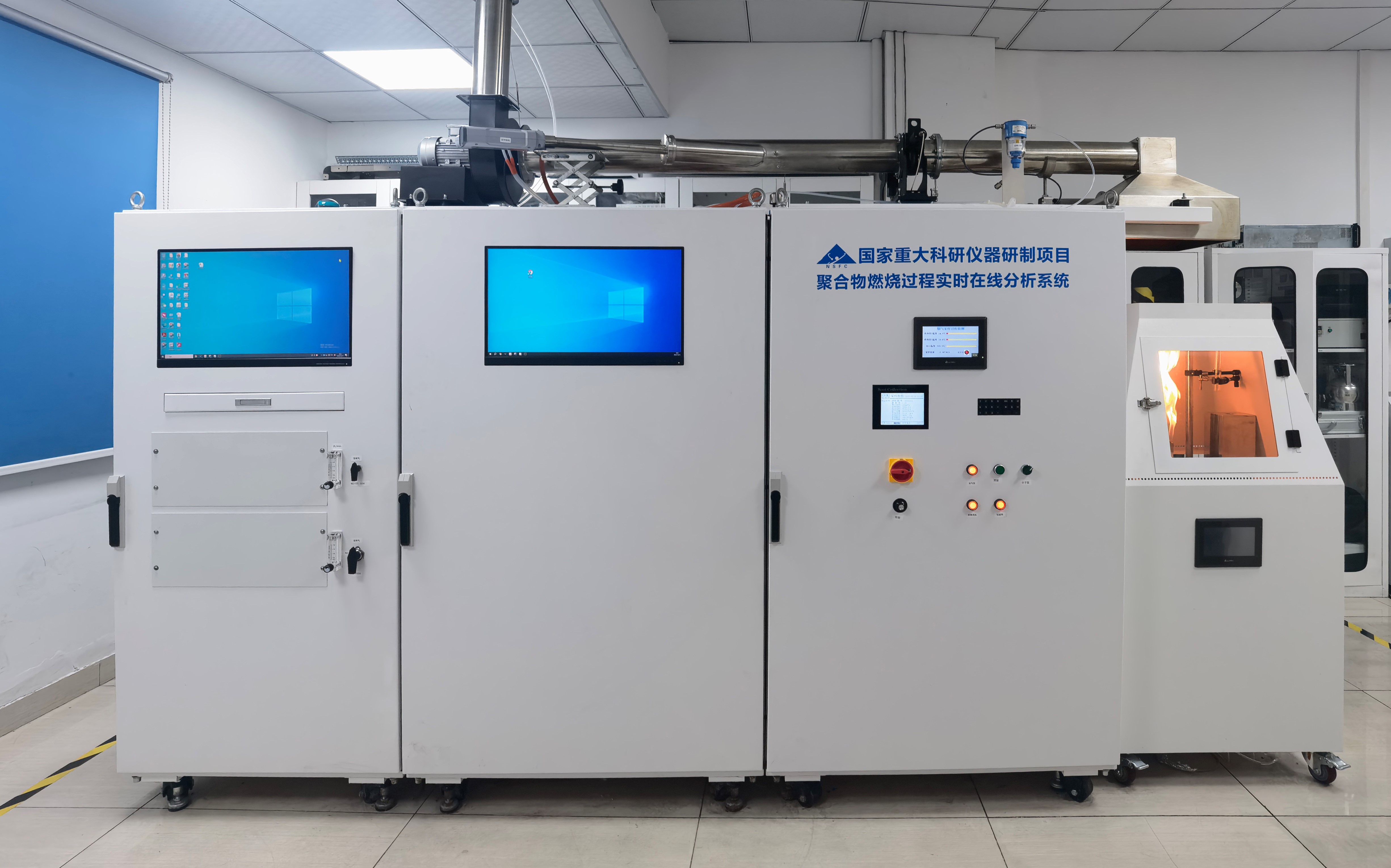

Supplement: Supplementary 1 — Figs. S1 to S9 Tables S1 to S8 [file research.0406.f1.zip › SI_figure/Figure S1.jpg]

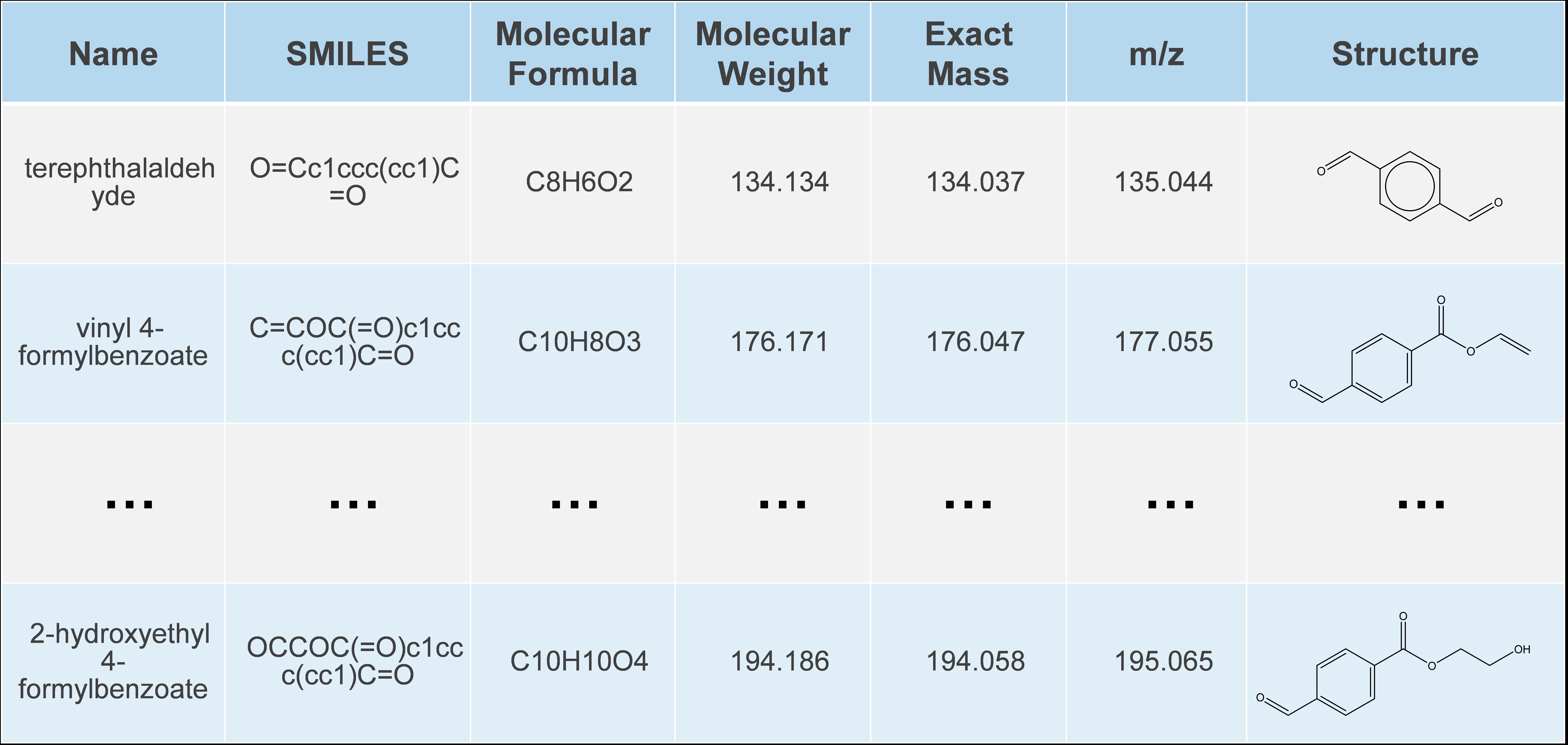

Supplement: Supplementary 1 — Figs. S1 to S9 Tables S1 to S8 [file research.0406.f1.zip › SI_figure/Figure S2.jpg]

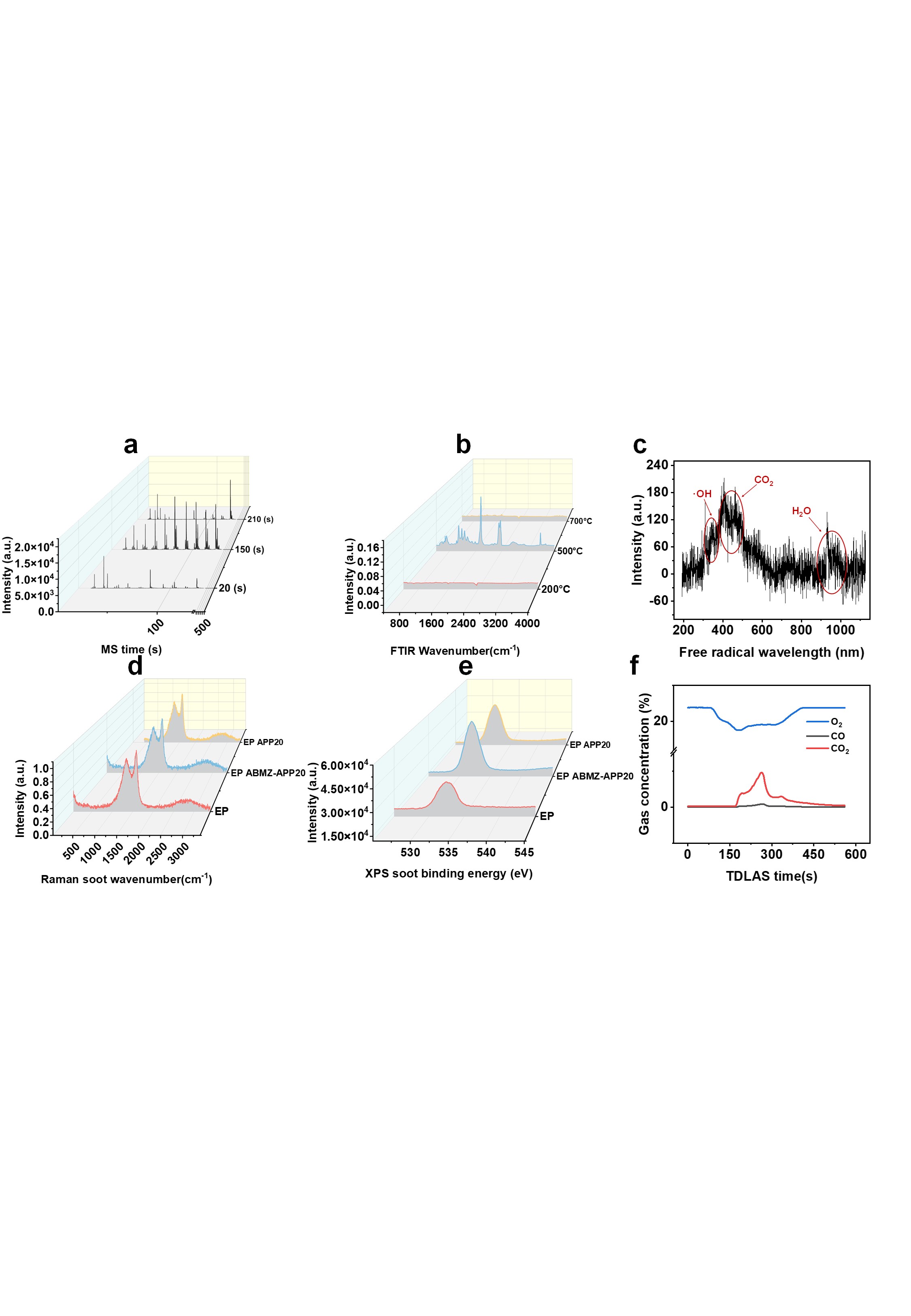

Supplement: Supplementary 1 — Figs. S1 to S9 Tables S1 to S8 [file research.0406.f1.zip › SI_figure/Figure S3.jpg]

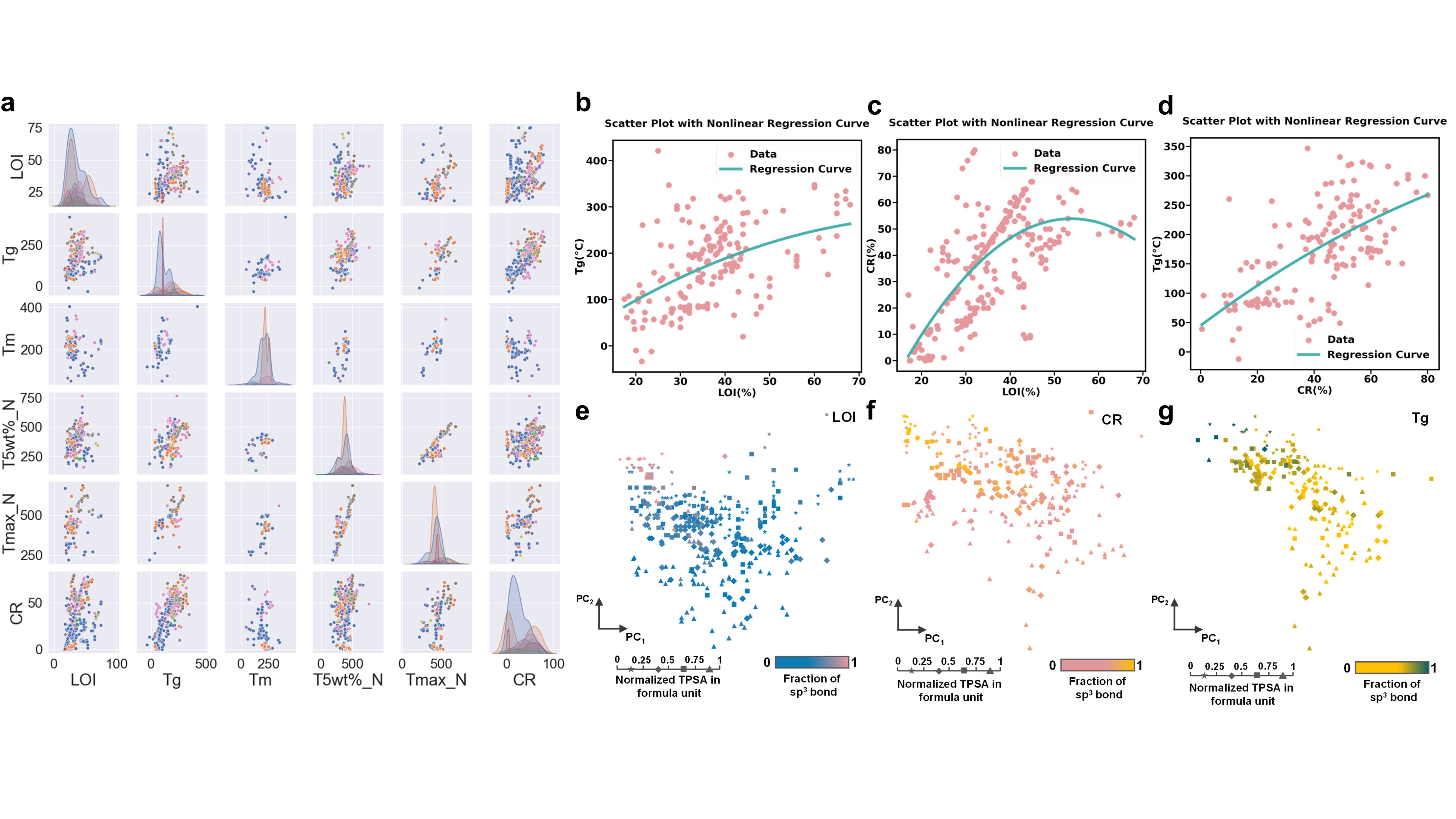

Supplement: Supplementary 1 — Figs. S1 to S9 Tables S1 to S8 [file research.0406.f1.zip › SI_figure/Figure S6.jpg]

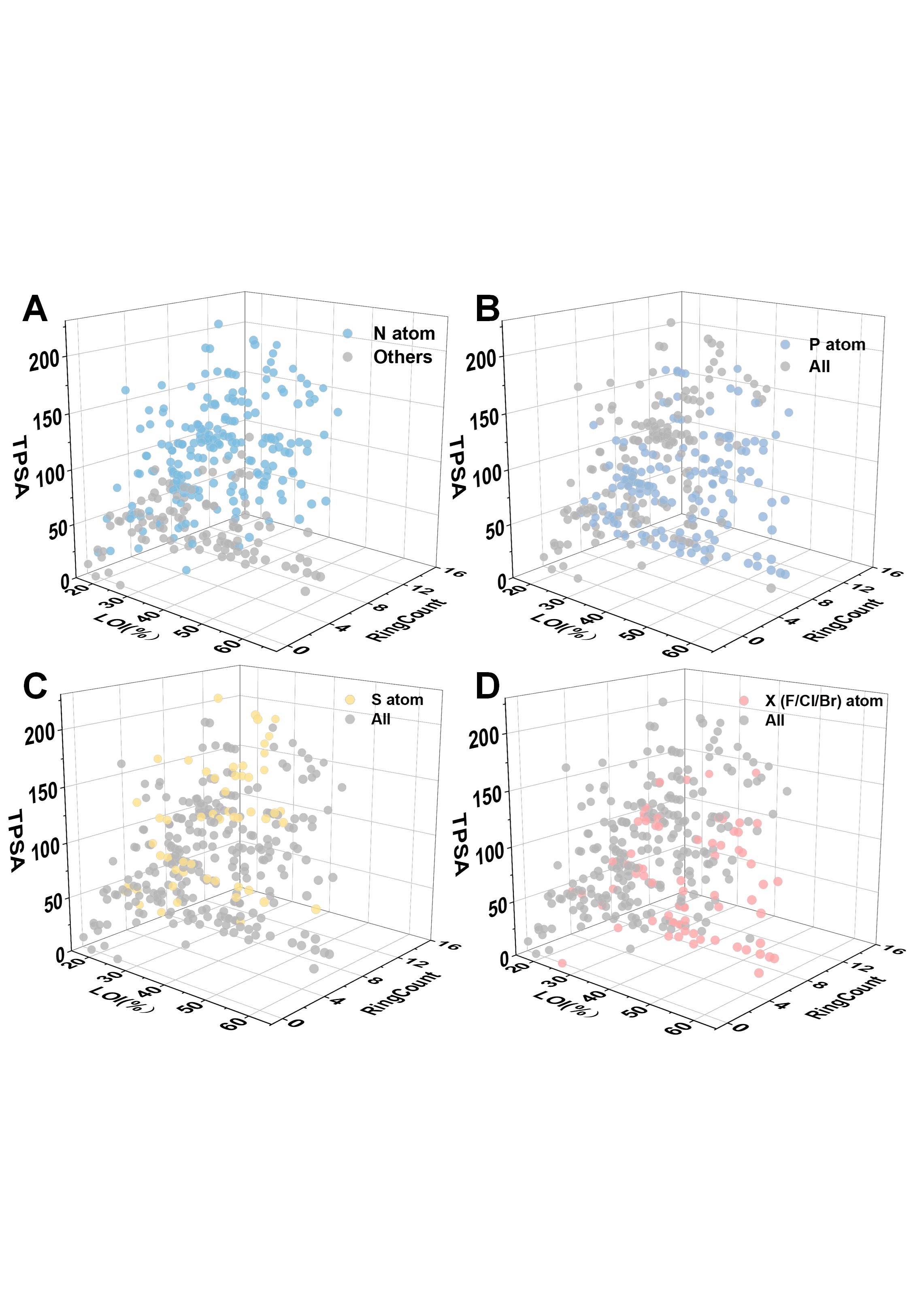

Supplement: Supplementary 1 — Figs. S1 to S9 Tables S1 to S8 [file research.0406.f1.zip › SI_figure/Figure S7.jpg]

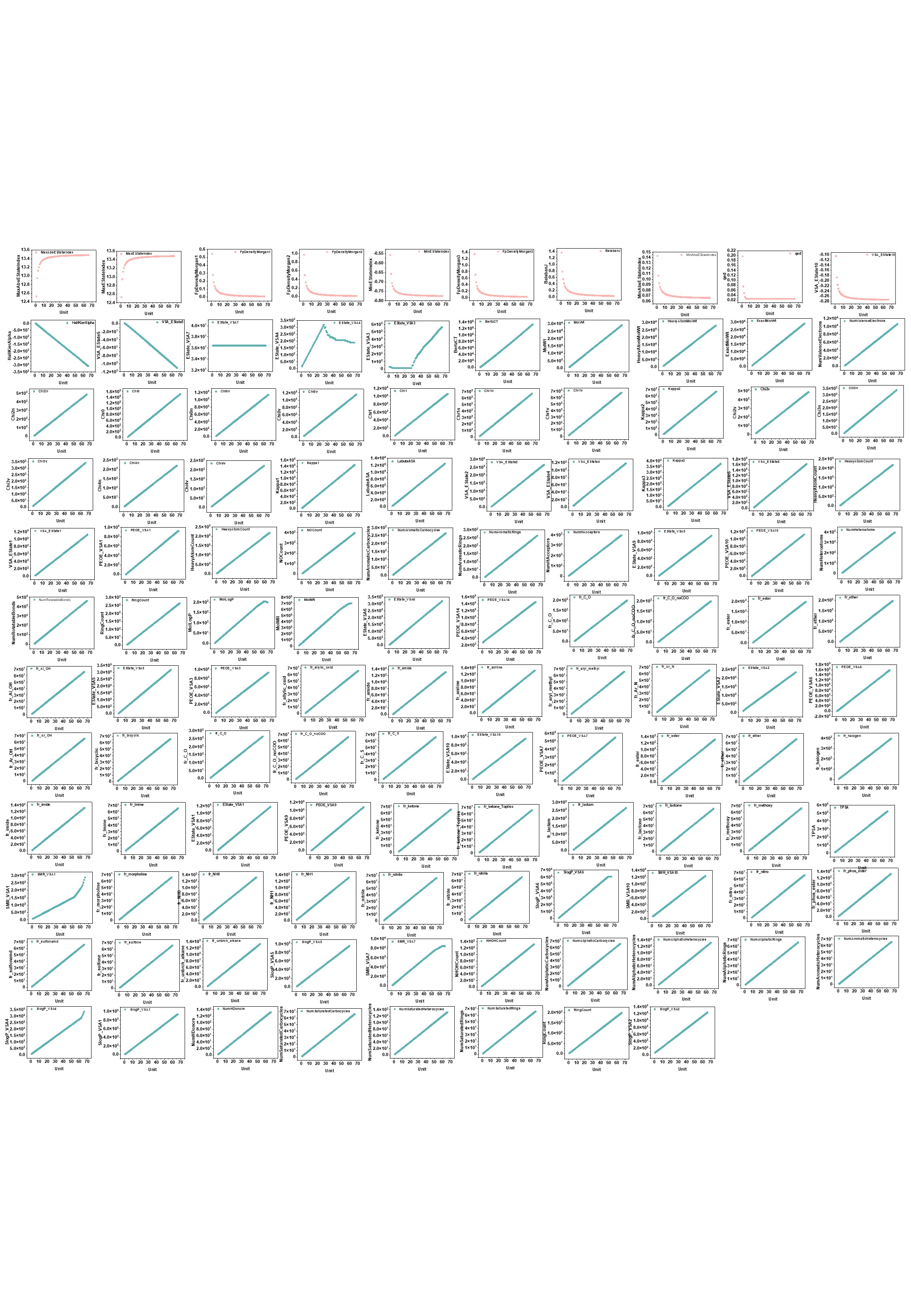

Supplement: Supplementary 1 — Figs. S1 to S9 Tables S1 to S8 [file research.0406.f1.zip › SI_figure/Figure S8.jpg]

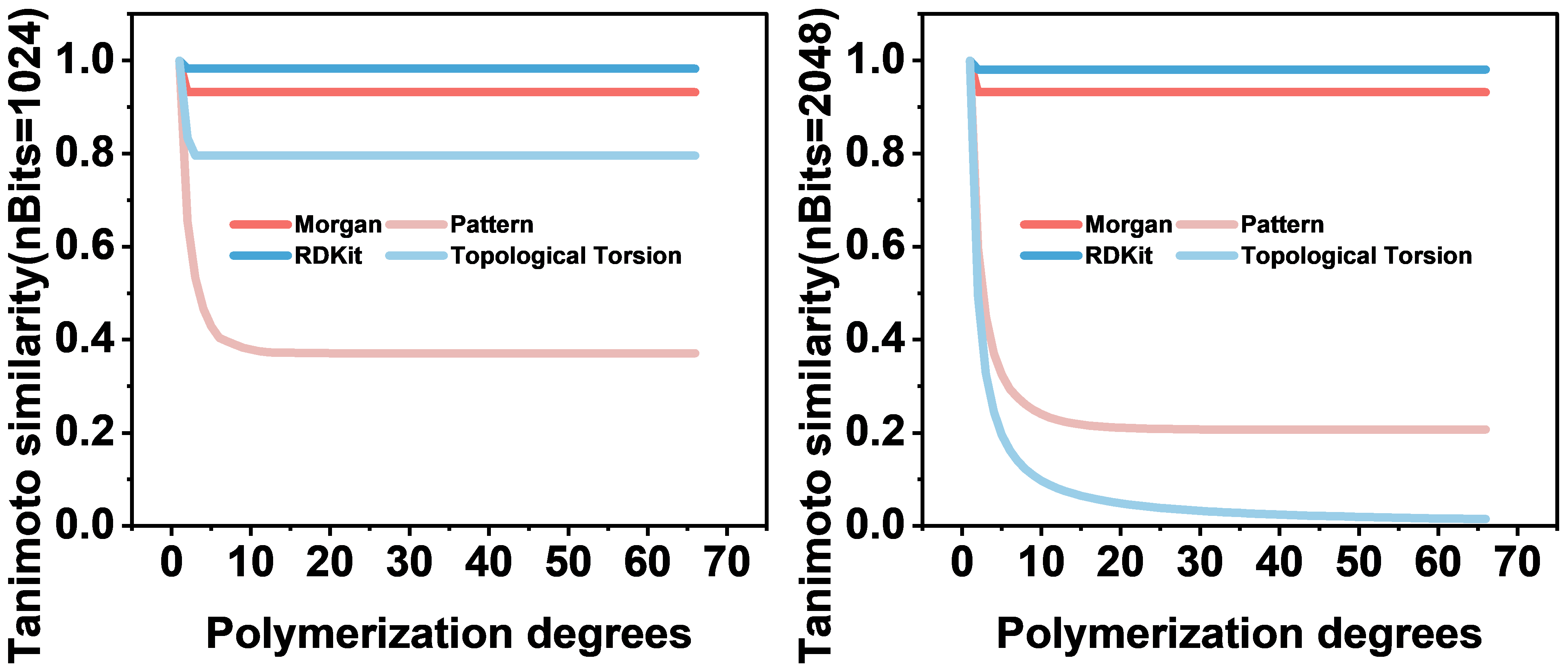

Supplement: Supplementary 1 — Figs. S1 to S9 Tables S1 to S8 [file research.0406.f1.zip › SI_figure/Figure S9.png]
